# Dislocation Based Flow Stress Model of 300M Steel in Isothermal Compression Process

**DOI:** 10.3390/ma11060972

**Published:** 2018-06-08

**Authors:** Rongchuang Chen, Peng Guo, Zhizhen Zheng, Jianjun Li, Fei Feng

**Affiliations:** State Key Laboratory of Material Processing and Die & Mould Technology, Huazhong University of Science and Technology, Wuhan 430074, China; crc@hust.edu.cn (R.C.); guop@hust.edu.cn (P.G.); jianjun@mail.hust.edu.cn (J.L.); fengfei@hust.edu.cn (F.F.)

**Keywords:** dislocation, stress-strain relationship, grain size, isothermal compression

## Abstract

The relationship between microstructure and flow behaviour has attracted attention from many researchers for the past decades, whilst the influences of dislocation and recrystallization on flow stress have not been well understood, which led to failure in flow stress prediction at high temperature compressions. In this work, we tried to provide a novel explanation of the relationship between microstructure evolutions and flow behaviour, and the influence of dislocation and recrystallization on flow stress was investigated. A dislocation based flow stress model was proposed and applied for 300M steel at the strain rate of 0.01–10 s^−1^ and the temperature of 950–1150 °C. Results showed the established model could predict the flow stress both at constant strain rate conditions and at variable strain rate conditions. The present investigation is helpful to a better understanding of hardening and softening mechanisms in hot compression of 300M steel.

## 1. Introduction

The 300M steel is an ultra-high strength steel, which is developed from 4340 steel by adding silicon and vanadium. It is the most commonly used material in manufacturing of aircraft landing gear, pressure vessels, and fasteners in aerospace, nuclear, and other fields because of its excellent overall performance. In manufacturing of the 300M steel heavy forgings, the material undergoes sophisticated deformation procedures, and a deep understanding of flow behaviour was critical for the quality control both at the macroscopic level and at the microscopic level.

Many researchers have made plenty of efforts on a deeper understanding of the relationship between the deformation behaviour and the microstructure evolution. The Johnson-Cook model [1], Arrhenius model, and its improved models [2] have been used in the modelling of stress-strain relationships of materials, such as 20Cr2Ni4A [3], stainless steel [4], aluminum alloy [5], and GCr15 [6]. Fuzzy neural network has also been used in constitutive modelling of Ni–Ti alloy [7], aluminium alloy [8], and Titanium alloy [9]. However, the underlying mechanism of the influence of microstructure evolution on flow stress was not established. Zerilli et al. [10] proposed Zerilli-Armstrong (ZA) model for face center cubic (FCC) materials based on the thermal activated dislocation motion theory, and the ZA model has been successfully used in high strength steel [11], 20CrMo [12], and Ti6Al4V alloy [13]. Internal state variable model [14,15], Kocks-Mecking (KM) model [16] and other models [17,18] were proposed to take into consideration of the influence of dislocation and recrystallization. But these models were not unified at different stages (the dynamic recovery stage and the dynamic recrystallization stage), and these models cannot be used in varying strain rates compressions.

In the present investigation, a unified flow stress model will be provided to describe the hardening and softening mechanisms of 300M steel in recrystallization. Hot compressions will be carried out on a thermal compression machine, and the flow stress model parameters will be obtained. The model will be verified both at constant strain rate compressions and at variable strain rate compressions.

## 2. Materials and Experimental Procedures

### 2.1. Materials

**Figure 1:**
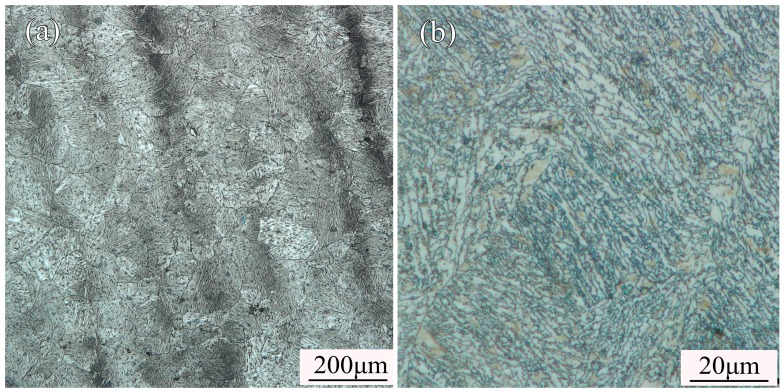
Microstructure of as-received 300M steel photographed on optical microscope showing: (**a**) the initial grains were equiaxed and (**b**) the initial phase was spheroidized pearlite.

The composition of the 300M steel is (weight percentage) 0.44% C–0.80% Cr–1.8% Ni–0.78% Mn–1.64% Si–0.35% Mo–0.06% V–balanced Fe. The initial state of the as-received material was annealed. The initial grains were equiaxed, and the initial phase was spheroidized pearlite, shown in Figure 1. The specimens were turned to cylindrical shape with the size of 8 mm in diameter and 12 mm in height.

### 2.2. Experiments

Thermal compressions were carried out on a thermal compression machine (Gleeble 3500, Dynamic Systems Inc., Austin, TX, USA). The samples were heated to 1200 °C at 200 °C/s, held for 180 s to homogenize their microstructures, and then cooled to specific temperatures. The samples were compressed at 4 different strain rates (0.01, 0.1, 1, and 10 s^−1^) and 5 different temperatures (950–1150 °C with 50 °C intervals) and quenched to maintain high temperature austenite grain boundaries. The true strain-stress curves were subject to noise reduction. The specimens were tempered at 560 °C for 2.5 h. After air cooling, they were wire-electrode cut along the symmetrical plane passing through the cylinder axis. Grinding, polishing, and etching was adopted to obtain the microstructure. The etching solution contained 5 mL of saturated picric acid, 2 mL of detergent, 2 mL of carbon tetrachloride, and 0.15 mL of hydrochloric acid. After corrosion at 25 °C, the center of the specimen was photographed using an optical microscope (VHX-1000C, Keyence Co., ‎Osaka, Japan). The average grain size of as-received 300M steel was 37.7 μm.

## 3. Results and Discussion

### 3.1. Deformation Behaviour

**Figure 2:**
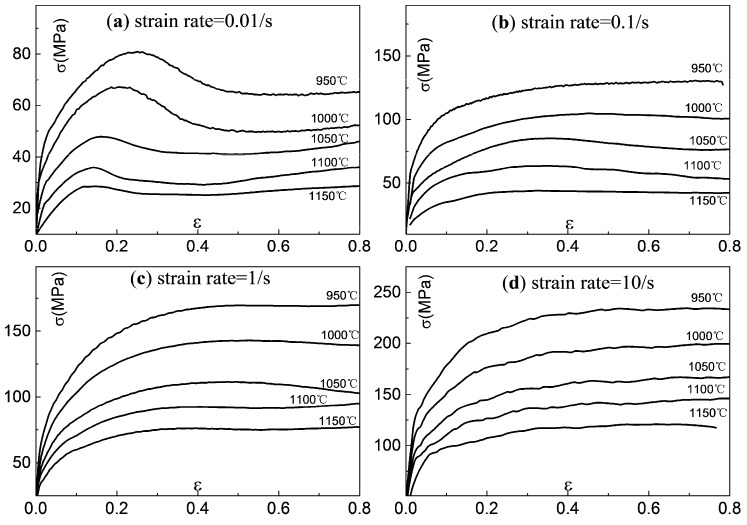
Stress-strain relationships of 300M steel compressed at different temperatures and strain rates: (**a**) 0.01 s^−1^, (**b**) 0.1 s^−1^, (**c**) 1 s^−1^, and (**d**) 10 s^−1^.

Stress and strain relationships of 300M steel compressed at various temperatures and strain rates are shown in Figure 2. At the starting stage, the stresses increased in proportion to the strains due to dislocation pile-ups and cross-slips. When the stored energy of deformation increased to a critical value, dynamic recrystallization (DRX) occurred. The recrystallized grains nucleated at grain boundaries where the dislocation densities were high. Under the driving force of grain boundary curvature and deformation energy, grain boundaries of recrystallized grains migrated [19] and as a result, the overall dislocation density decreased, which led to the flow stress decreasing at the recrystallization stage. Then the work hardening effects and softening effect finally came to a balance [20] as the flow stress remained approximately unchanged.

As can be seen, the microstructure evolution played a very important role in flow behaviour of 300M steel, but yet very few models can be implemented which take into consideration of the influences of dislocation and recrystallization on flow stress. Therefore, a model accurately describing the flow behaviour of 300M steel is urgently needed.

### 3.2. Microstructures Evolution

**Figure 3:**
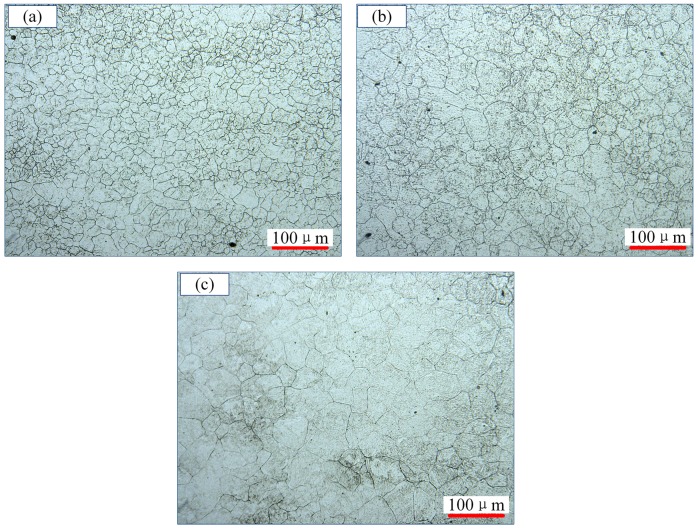
Microstructures of 300M steel deformed at 10 s^−1^ at: (**a**) 1050 °C; (**b**) 1150 °C; and (**c**) 1200 °C.

**Figure 4:**
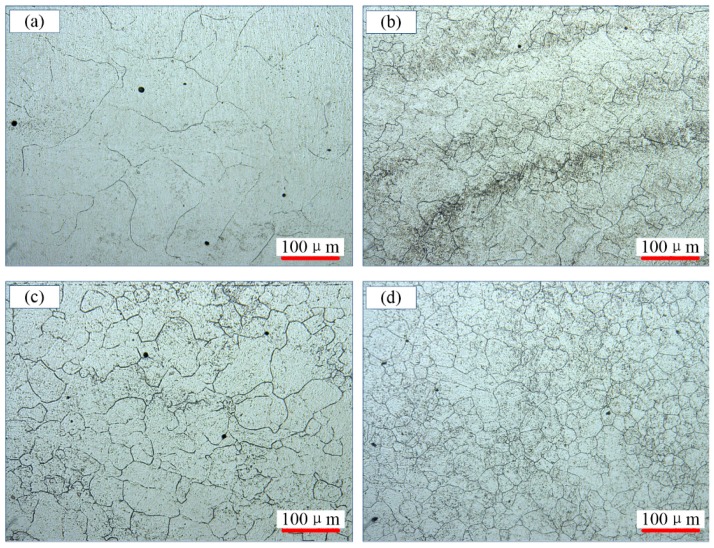
Microstructures of 300M steel deformed at the strain rate of: (**a**) 0.01 s^−1^; (**b**) 0.1 s^−1^; (**c**) 1 s^−1^; and (**d**) 10 s^−1^.

The microstructure evolutions of 300M steel were investigated via etching, showing in Figure 3 and Figure 4. At strain rate of 10 s^−1^ in Figure 3, the average grain sizes were 16.1, 27.8 and 42.2 μm, respectively. The grains were significantly refined at 1050 °C (Figure 3a) and almost all grains were recrystallized. It was interesting that recrystallizations did not refine the grains in Figure 3c, but led to grain coarsen at high temperatures, which was due to grain growth after recrystallization was completed, as could be seen from Figure 3c that a few abnormal growth grains with size of ~90 μm emerged.

Figure 4 shows the microstructures of 300M steel deformed at 1150 °C to a strain of 1.2. The average grain sizes were 78.0, 52.4, 46.2 and 42.2 μm, respectively. The grains were finer deformed at a higher strain rate, as seen in Figure 4a,d. This could be explained by that the recrystallization and grain growth took place simultaneously during compression, and if the strain rate was too small, the grain refinement effect by recrystallization could be reduced or overcome by the grain coarsen effect by grain growth.

The microstructure evolution could be expressed by a volume fraction (*X*) and the average grain size (*d*), which represented the progress of recrystallization and grain size variation in compression. The volume fraction *X* could be expressed as [21]:

(1) $$X=1-e^{-0.693{\left(\frac{\varepsilon -\varepsilon_{c}}{\varepsilon_{p}}\right)}^{n}}$$

where $\varepsilon_{c}$ was the recrystallization critical strain, $\varepsilon_{p}$ was the peak strain of the stress-strain curve, and *n* was a constant related to the rate of dynamic recrystallization. The values of $\varepsilon_{c}$ and $\varepsilon_{p}$ could be obtained from the stress-strain curves and modelled as follows:

(2) $$\varepsilon_{c}=0.02738\cdot {\left(\dot{\varepsilon }\cdot e^{\frac{379800}{RT}}\right)}^{0.04628}$$

(3) $$\varepsilon_{p}=0.0326\cdot {\left(\dot{\varepsilon }\cdot e^{\frac{379800}{RT}}\right)}^{0.0602}$$

The constant *n* was determined to be 1.703 through regression of experimental values according to Equation (1).

**Figure 5:**
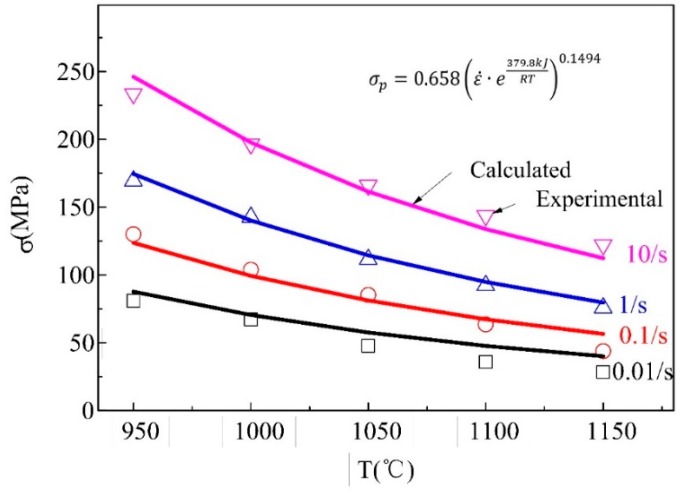
Fitting of peak stress to obtain the activation energy.

The complete recrystallization grain sizes at different temperatures and strain rates could be obtained via metallography. The relationship between recrystallization grain sizes ($d_{drx}$) and deforming conditions represented by Zener-Hollomon parameter (*Z*), in which the strain activation energy was calculated to be 379.8 kJ/(mol K), is shown in Figure 5.

Therefore, the recrystallization grain sizes ($d_{drx}$) was:

(4) $$d_{drx}=6568.2\cdot {\left(\dot{\varepsilon }\cdot e^{\frac{379800}{RT}}\right)}^{-0.159}$$

and the average grain size (*d*) was expressed as:

(5) $$d=Xd_{drx}+\left(1-X\right)d_{0}$$

where $d_{0}$ represented the initial grain size, which was 37.7 μm determined by metallography.

### 3.3. Dislocation Based Flow Stress Model

The flow stress was closely related the dislocation motions, as described by the Taylor’s equation [22,23,24].

(6) $$\sigma =\alpha \mu Mb\sqrt{\rho }$$

where σ was the flow stress, α a constant, μ the shear modulus, M the Taylor factor, b the Burgess vector, and ρ the average dislocation density. In the recovery stage, the dislocation density increment (Δρ) could be described by the contradicting effects of the strain hardening ($\Delta \rho_{s}^{+}$) which was caused by dislocation pile-ups and cross-slip, and the softening ($\Delta \rho_{s}^{+}$) by creep and dynamic recovery dislocation annihilations by the Kocks-Mecking (KM) model [16]:

(7) $$\Delta \rho =\Delta \rho_{s}^{+}-\Delta \rho_{drv}^{-}$$

where $\Delta \rho_{s}^{+}$ was dislocation increment by strain hardening, and $\Delta \rho_{drv}^{-}$ was dislocation decrement by dynamic recovery, which followed [25,26]:

(8) $$\Delta \rho_{s}^{+}=\frac{M}{b}\left(\frac{\sqrt{\rho }}{k_{1}}+\frac{1}{d_{0}}\right)\Delta \varepsilon_{eff}^{p}\;\mathrm{and}\;\Delta \rho_{drv}^{-}=k_{2}\rho \Delta \varepsilon_{eff}^{p}$$

where $d_{0}$ was the initial grain size, $k_{1}$ and $k_{2}$ coefficients, and $\Delta \varepsilon_{eff}^{p}$ the effective plastic strain increment. It was argued by researchers [27,28] that the dislocation-free recrystallized nuclei began to form and grow at grain boundaries under the driving force of grain boundary curvature and dislocations during dynamic recrystallization. But the dislocation model did not take into consideration of the dislocation decrement by dynamic recrystallization, which led to an inaccuracy in flow stress calculation. Therefore, the following model was proposed:

(9) $$\Delta \rho =\Delta \rho_{s}^{+}-\Delta \rho_{drv}^{-}-\Delta \rho_{drx}^{-}+\Delta \rho_{g}^{+}$$

where the $\Delta \rho_{drx}^{-}$ represented the dislocation decrement by dynamic recrystallization, and $\Delta \rho_{g}^{+}$ was introduced to take into consideration the Hall-Petch effect, which needed to be determined.

Dislocation annihilation due to recrystallization was nonnegligible for low stacking fault energy (SFE) materials, such as 300M steel (11.4 mJ/m^2^), because for low SFE materials, the mobility of dislocations was lower, and the dynamic recrystallization was more likely to occur due to dislocation pile-ups in local areas [27]. As the recrystallized grains nucleated and grew at grain boundaries, the average dislocation densities of boundary swept regions decreased [28,29], and the $\rho_{drx}^{-}$ was reasonably proportional to dynamic recrystallization volume fraction (*X*) until the recrystallization completed. It could be assumed that the $\rho_{0}$ was the average dislocation densities of recrystallized grains as well as the as-received material, which was negligible compared with $\rho_{c}$, the critical dislocation density for recrystallization. The value of $\rho_{drx}^{-}$ followed:

(10) $$\rho_{drx}^{-}=k_{3}X\rho_{c}.$$

The differentiated form obeyed:

(11) $$\Delta \rho_{drx}^{-}=k_{3}\frac{\partial X}{\partial \varepsilon }\rho_{c}\Delta \varepsilon_{eff}^{p}.$$

In this way, the value of $\Delta \rho_{g}^{+}$ remained to be determined in Equation (9). Dynamic recrystallization influenced the flow stresses not only by dislocation annihilation but also by grain sizes variation, as shown in researches [30,31] that the relationship between flow stress and average grain size obeyed the Hall-Petch equation, to which the $\Delta \rho_{g}^{+}$ followed a similar expression and the incremental form shows as follows:

(12) $$\rho_{g}^{+}=\frac{k_{4}}{bd}$$

thus,

(13) $$\Delta \rho_{g}^{+}={\dot{\rho }}_{g}^{+}\Delta \varepsilon_{eff}^{p}=-\frac{k_{4}}{bd^{2}}\frac{\partial d}{\partial \varepsilon }\Delta \varepsilon_{eff}^{p}$$

where $k_{4}$ was a coefficient, and b the Burgess vector. Although previous models [28,29,30,31] can separately calculate the flow stress in the dynamic recovery stage or dynamic recrystallization stage, no unified flow stress model covering the whole deformation stage has been reported. However, in the present model, at the dynamic recovery stage, both $\Delta \rho_{drx}^{-}$ and $\Delta \rho_{g}^{+}$ equal 0, and it was consistent with Kock-Mecking model. In recrystallization, the dislocation annihilation and grain size effect were incorporated. The form of the model was unified at different stages.

### 3.4. Model Parameter Determination

The coefficients of the flow stress model (*k*_1_–*k*_4_) could be expressed in the Arrhenius type:

(14) $$k_{i}=A_{i}{\left(\dot{\varepsilon }\cdot e^{\frac{Q_{i}}{RT}}\right)}^{n_{i}}$$

where *i* equaled 1–4 to represent *k*_1_–*k*_4_. The model parameters ($A_{i},\;n_{i},\;Q_{i}$) could be determined in an iterative solution. The dislocation density for the *i*+1th iteration was:

(15) $$\rho_{i+1}=\rho_{i}+\frac{M}{b}\left(\frac{\sqrt{\rho_{i}}}{k_{1}}+\frac{1}{d_{0}}\right)\Delta \varepsilon_{eff}^{p}-k_{2}\rho_{i}\Delta \varepsilon_{eff}^{p}-k_{3}\rho_{c}\frac{\partial X}{\partial \varepsilon }\Delta \varepsilon_{eff}^{p}-\frac{k_{4}}{bd^{2}}\frac{\partial d}{\partial \varepsilon }\Delta \varepsilon_{eff}^{p}$$

where $\rho_{i}$ was the dislocation density of the *i*th iteration, and $\Delta \rho_{i}$ the increase of dislocation, the value of which was obtained via experiment. The other parameters are shown in Table 1. The critical dislocation densities for dynamic recrystallization was obtained via fitting:

(16) $$\rho_{c}=9.5479\times 10^{8}{\left(\dot{\varepsilon }\cdot e^{\frac{463520}{RT}}\right)}^{0.2362}.$$

**Figure 6:**
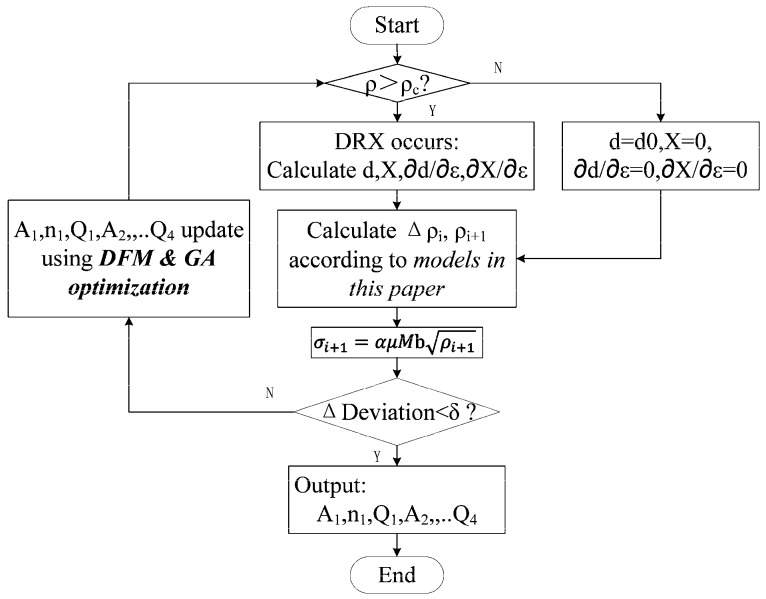
Iterative solving procedure.

The iterative solving, which was similar to multi-parameter nonlinear regression process, was implemented in MATLAB and the procedure is shown in Figure 6. Starting from a set of initial values, the flow stresses were calculated, and the model parameters were optimized to minimize the deviations using an algorithm which was a combination of derivative free method (DFM) and genetic algorithm (GA). The iterative solving results were shown in Table 2.

### 3.5. Verification and Comparison

**Figure 7:**
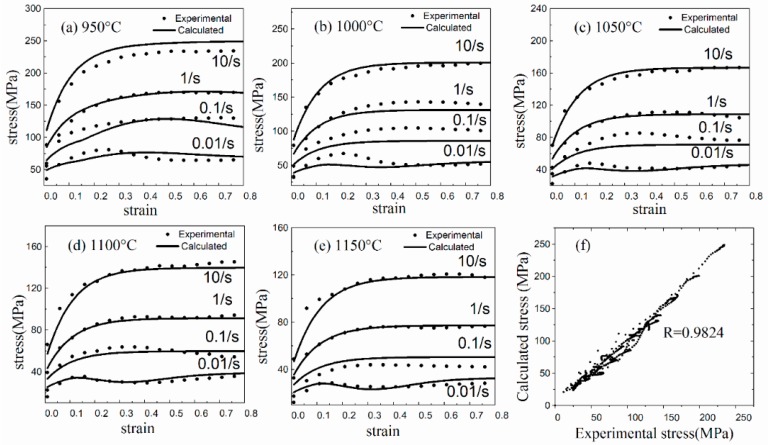
Comparison of experimental and calculated flow stress of 300M steel. (**a**) 950 °C, (**b**) 1000 °C, (**c**) 1050 °C, (**d**) 1100 °C, (**e**) 1150 °C, and (**f**) a scatter plot drawn from experimental and calculated stresses.

Figure 7 shows the comparison of flow stress of 300M steel obtained by experiment and model calculation. The mean absolute and relative deviation were 5.37 MPa and 6.59%, respectively. A scatter plot drawn from experimental and calculated stresses (Figure 7f) showed that the model was accurate, and the R value in representative of the confidence level was calculated to be 0.9824. Minor deviations between the calculated and experimental flow stresses were found at low strain rates, which could be attributed to two reasons. Firstly, the optimization algorithm in this investigation tended to minimize the mean deviation of all flow stresses, resulting in a bigger percentage deviation at low strain rates. Secondly, some errors in experimental data may be caused by the drum effect of deformed specimens, the uneven temperature distribution, and the friction at both ends of the specimens. The Modified Arrhenius [2], Kock-Mecking [16], and Johnson-Cook [1] models were also employed, and the confidence levels were calculated to be 0.977, 0.978 and 0.972, which showed that the model in this investigation has a slight advantage in precision. Besides, the model has successfully described the relationship between metal flow behaviour and microstructure evolution.

**Figure 8:**
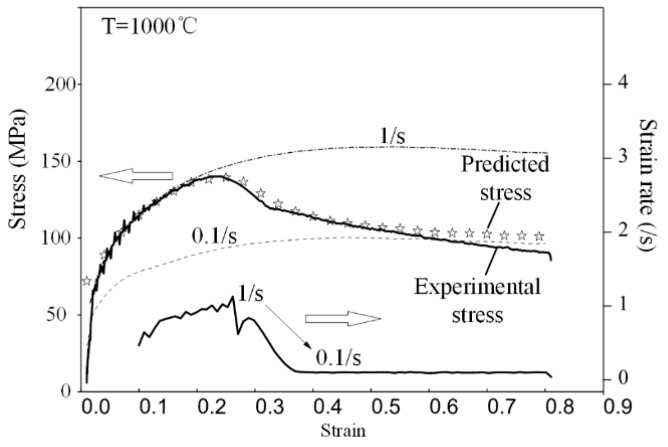
The measured strain rate, the predicted flow stress, and the measured flow stress of a variable strain rate compression.

In the final step, the model was adopted in flow stress prediction of a variable strain rate compression. The experimental strain rates varied from 1 to 0.1 s^−1^ whilst the temperature remained unchanged. The measured strain rate, the predicted flow stress, and the measured flow stress in the experiment were shown in Figure 8. The predicted flow stress agreed well with the experiment value. Therefore, the flow stress model proposed in this investigation was able to precisely calculate flow stress of 300M steel in variable strain rate compressions.

## 4. Conclusions

The flow behaviour and microstructure evolution of 300M steel was investigated via isothermal compression at the strain rate of 0.01–10 s^−1^ and the temperature of 950–1150 °C, and the following conclusions can be drawn:

(1) A dislocation based flow stress model was proposed to take into consideration of dynamic recrystallization and Hall-Petch effect. The model was unified both at the recovery stage and at the recrystallization stage.
(2) Microstructure evolution in dynamic recrystallization of 300M steel was established via metallography. The dynamic recrystallization of 300M steel was described in terms of the dynamic recrystallization volume fraction model and the average grain size model, which were determined by fitting.
(3) Flow stress model parameters were obtained by an iterative procedure implemented in Matlab software. A comparison between predicted and experimental flow stresses both at constant strain rate compressions and at variable strain rate compressions was made, and results showed a high precision in flow stress prediction of 300M steel.

## Figures and Tables

**Table 1:** Model parameters.

Parameters	Value	Unit	Ref.
α	0.3	—	[22]
μ	86.94 − 0.027T	GPa	[23]
*M*	3.06	—	[22]
*b*	2.54 × 10^−10^	m	[24]

**Table 2:** Iterative solving results.

Parameters	$A_{i}$	$n_{i}$	$Q_{i}$
$k_{1}$	5.87 × 10^3^	−1.83 × 10^−1^	2.66 × 10^5^
$k_{2}$	1.35 × 10^1^	−1.60 × 10^−3^	−2.42 × 10^6^
$k_{3}$	1.19 × 10^−3^	−2.88 × 10^0^	2.17 × 10^4^
$k_{4}$	6.33 × 10^−43^	−4.95 × 10^−1^	−2.28 × 10^6^
